# Abstract of Proceedings of the Buffalo Medical Association

**Published:** 1866-12

**Authors:** T. M. Johnson

**Affiliations:** Sec’y.


ART. II.—*Abstract of Proceedings of the Buffalo Medical Association.*

TUESDAY EVENING, NOV. 6th, 1866.

The Association was called to order at the usual hour, Dr. H. M. Congar in the Chair. Present—Drs. Miner, Congar, Smith, Garvin, Cronyn, Gleason, Gay, J. R. Lothrop, Rochester, Ring and Johnson. The minutes of the last meeting were read and adopted.

Dr. Thomas R. Lothrop was, by vote of the Association, admitted to membership.

Dr. Augustus Mackey was, by vote of the Association, invited to be present at its meetings until he becomes eligible to membership.

DR. ROCHESTER reported the following case: A lady who had been in delicate health for several months previous, and had during the two months immediately preceding her last illness been suffering from constipation and hemorrhoides, ate quite heartily of oysters on Sunday evening, and was early Monday morning taken with vomiting and purging, which continued until Tuesday morning, when I first saw her. She had then had twenty evacuations of the bowels. Saw her again on Tuesday at half past one P. M., and again at three P. M. In this interval she had five choleraic or rice-water evacuations of the bowels and severe vomiting. Wednesday evening she passed into collapse. She took two bottles of champagne during Wednesday night, which seemed to control the vomiting. Urinary secretion was entirely suspended during her illness. Passed a catheter several times without obtaining any urine. She died on Thursday. The immediate cause of death was the extreme exhaustion produced by the disease.

The same evening a servant girl in the house was taken with a severe diarrhœa which was very persistent. On Sunday evening following a boy was brought from a canal-boat to the Sisters of Charity Hospital with severe choleraic purging and vomiting.

Dr. Miner said that it seemed remarkable that cholera should prevail to so great an extent in most of the larger cities of the country and so few cases be reported in Buffalo. This case, occurring in the locality it did, is indeed singular. If eating oysters or any other impropriety in eating was the cause, it will show the close connection between cholera and cholera morbus. Have seen a case within the month on South Division street of a child fourteen months old who was taken in the night with cholera morbus. In the morning was pale and prostrated, and at night went into collapse and died.

Saw also another case of a child who had frequent watery stools through the day and was weak and prostrated through the night, and the next morning after taking its usual food laid back upon its pillow and died.

These cases are such as we have seen every year since cholera prevailed. I think that when there is no cholera prevailing physicians call such cases cholera morbus. When cholera is prevailing well-informed physicians probably call many cases cholera that are cholera morbus.

That the patient mentioned by Dr. Rochester had the usual symptoms of cholera in severe and fatal form cannot be doubted, but since it was an isolated case and lacked the exposure to infection from choleraic disease, we cannot in accordance with the prevailing code concerning this disease willingly adopt the idea of Asiatic cholera in Buffalo.

Dr. Rochester said that it was as clear a case of cholera as he ever saw. It may have been the first and only case. I called it a case of sporadic cholera. I have often seen cases of sporadic cholera, and seen them prove fatal.

Dr. Gay reported the case of a lady residing on Vine street who had all the usual symptoms of cholera, viz: rice-water evacuations, vomiting, complete suppression of urinary secretions, etc. Gave in this case byhypodermic injection two grains of the sulphate of morphine within an hour, which controlled the vomiting and in great degree mitigated all the symptoms of the disease. Had also another case of a lady residing on Chippewa street, about forty years of age, taken suddenly with cholera and suffered severely, but this case like the first mentioned terminated favorably.

Dr. Miner said that the cases reported by Drs. Rochester and Gay were interesting, and are valuable as evidence in regard to the communicability of cholera.

Dr. Garvin said that in July last he was called about two o'clock in the morning to see a lady about forty years of age, who was suffering from severe cramps, rice-water evacuations and vomiting. Gave injections of starch-water and tinct. opii, followed with hydg. cum creta and opiates. Voided no urine for twelve hours; did not use a catheter. Patient convalesced favorably.

Scarlatina, rubeola and pneumonia were the diseases mentioned as prevailing.

Adjourned. T. M. Johnson, Sec'y.

